# UAV-based application of fungicides for management of blast disease in rice: comparative evaluation with manual spraying at recommended and reduced doses

**DOI:** 10.3389/fpls.2026.1936875

**Published:** 2026-10-30

**Authors:** Talluri Kiran Babu, N. Rama Gopala Varma, A. Ramakrishna, G. Shiva Prasad, N. Lingaiah

**Affiliations:** 1 Rice Research Unit, Agricultural Research Institute, Professor Jayashankar Telangana Agricultural University, Rajendranagar, Hyderabad, Telangana, India; 2 Agricultural Research Station, Kampasagar (PJTAU), Nalgonda, Telangan, India

**Keywords:** drone spraying, fungicide dose optimization, *Magnaporthe oryzae*, precision crop protection, rice blast, uncrewed aerial vehicles (UAVs)

## Abstract

Rice blast, caused by *Magnaporthe oryzae*, is a widely distributed and most destructive disease in rice cultivation, threatening global food security. Farmers are increasingly adopting Uncrewed Aerial Vehicles (UAVs) for pesticide application in rice with reduced doses. However, scientific evidence-based fungicide dose reduction under UAV-assisted spraying remains limited in India. A field experiment was conducted over two crop growing seasons (*kharif*, 2022 and *rabi*, 2022-23) at one location to evaluate the control efficacy of two combination fungicides *i.e.*, Azoxystrobin + Tricyclazole, Azoxystrobin + Difenoconazole, and one regularly used fungicide, Tricyclazole 75% WP against blast, applied through UAV’s at recommended (100% @ 1X) and reduced dosages (75% @ 0.75X) and compared with their corresponding application using the knapsack power sprayer (100% @ 1X). Disease severity, control efficacy, grain yield, economic returns, and phytotoxicity were assessed under field conditions during both the seasons. The application of conventional fungicides using UAV at the recommended dosage (1X) consistently exhibited better control of leaf and neck blast than the corresponding reduced-dose UAV treatments (0.75X) and conventional knapsack power spraying (1X), with the effect is being more pronounced against neck blast under field conditions. Of the fungicides, Azoxystrobin 16.7% + Tricyclazole 33.3% SC and Azoxystrobin 18.2% w/w + Difenoconazole 11.4% w/w SC applied through UAVs at 1X dosage recorded the highest disease control efficacy, grain yield, and economic returns. This study represents one of the first systematic preliminary field evaluation of fungicide dose optimized under UAV-assisted spraying in India using currently registered conventional formulations without adjuvants while maintaining the active ingredient dose (*g a.i*. ha^-^¹).

## 1 Introduction

Rice (*Oryza sativa* L.) is a staple food for more than 50% of the global population, providing over 20% of dietary calories and playing a pivotal role in maintaining international food security, especially in Asia (Fukagawa and Ziska, 2019). Global rice consumption is projected to increase from 480 million tonnes in 2014 to nearly 550 million tonnes by 2030, driven by both population growth and economic development in emerging nations (Yuan et al., 2021). India is the world’s largest rice-producing country, contributing to nearly 28% of global rice production (FAOSTAT, 2025). However, sustaining this level of production is becoming increasingly challenging due to continuously evolving constraints. Nonetheless, rice cultivation is increasingly constrained by climate variability, water scarcity, declining soil fertility, salinity, emerging pest outbreaks, labor scarcity, soaring labor wages, *etc*. The increasing migration of agricultural workers to non-agricultural sectors is leading to a 12% decline in the workforce (Srivastava et al., 2020; Avhale et al., 2025), equivalent to nearly 30.7 million laborers (Subramanian et al., 2021). Consequently, farm operations are becoming increasingly expensive due to rising agricultural labor wages (Vaishnavi and Manisankar, 2022), ultimately affecting the productivity, profitability, and long-term sustainability of rice cultivation.

Among the major biotic stresses affecting rice, blast disease caused by *Magnaporthe oryzae* is regarded as one of the most destructive disease worldwide, causing significant yield losses ranging from 10-30% under favorable environmental conditions. In comparison, severe epidemics resulted in yield penalties exceeding 50%, depending on disease severity and the crop growth stage at the time of infection (Dean et al., 2012; Howard et al., 2025; Xu et al., 2026). The pathogen affects rice crop across all developmental phases, from the nursery to the grain-filling stage, with neck blast being particularly destructive due to its direct impact on grain development and yield (Xu et al., 2026). Globally, annual rice yield losses caused by blast were estimated to be sufficient to feed nearly 60 million people (Valent, 2021).

Chemical control with fungicides remains one of the most reliable approaches for managing rice blast, particularly under high disease pressure, in which many high-yielding cultivars are susceptible to rapidly evolving pathogen populations (Mohiddin et al., 2021). Historically, Tricyclazole has served as a benchmark fungicide for rice blast management owing to its high efficacy against *Magnaporthe oryzae* (Kumar et al., 2024). However, its prolonged and repeated use has raised concerns about fungicide resistance and pesticide residue accumulation in grains, leading to restrictions on its use in Basmati-growing and export-sensitive regions (Kumar et al., 2024). This has fostered the identification of newer combination fungicides for blast management. In this context, combination molecules involving strobilurin and triazole groups have emerged as promising alternatives in India due to their broad-spectrum activity and systemic properties (Mohiddin et al., 2021).

While chemical prophylaxis remains crucial, conventional ground-based spraying methods, such as knapsack and taiwan sprayers, are constrained by operational inefficiencies, walking difficulty, and postural discomfort (Ghugare et al., 1991) in the flooded rice ecosystem. These methods limit operator mobility in the field, resulting in large spraying volumes, coarse nozzles, and low spray quality, resulting in uneven spray deposition, substantial pesticide losses, which often expose farmers to toxic chemicals (Damalas and Koutroubas, 2016; Liu et al., 2025a; Wu et al., 2025). Consequently, these labor-intensive equipment types are no longer suitable for applying crop protection chemicals in rice (Pathak et al., 2022). Globally, pesticide use is associated with over three million poisoning cases, 2,20,000 deaths, and 7,50,000 chronic illnesses annually, predominantly in developing countries (Tudi et al., 2022).

The use of UAVs for pesticide application has seen unprecedented growth in the Indian rice production system over the past 5 years. It has gained increasing attention for its ability to manage insect-pests and diseases effectively and efficiently without damaging crop plants (Dou et al., 2022). Several studies reported improved efficacy compared to conventional spraying techniques (Qin et al., 2016; Subramanian et al., 2021; Varma et al., 2022; Ragiman et al., 2024; Yallappa et al., 2025). In this background, Ministry of Agriculture & Farmers Welfare, Government of India has released crop specific Standard Operating Protocols (SOPs) on use of drones for spraying of pesticides to accelerate mechanization in crop protection (Ministry of Agriculture & FW, GOI, 2023). The liberalized Drone Rules, 2021 are more permissive and are expected to remove unnecessary operational barriers to drone use in agriculture and to create a strong drone ecosystem in the country. According to statistics available on the Digital Sky portal of the Directorate General of Civil Aviation (DGCA), Government of India, a total of 174 type-certified drone models, 42419 registered Unique Identification Numbers (UINs), and 50976 certified remote pilots have been registered in India (https://www.dgca.gov.in). Notably, nearly 75% of certified drone models are agricultural UAVs specifically developed for precision pesticide application.

Despite these advancements, drone-based pesticide applications for several field crops are often applied at only 75% of the recommended dosage, without adjuvants, resulting in reduced spray volumes in the field. Such practices may compromise pesticide efficacy and spray performance, thereby creating doubts among farmers regarding the precision and effectiveness of this emerging technology. Notably, the long-term application of reduced fungicide concentrations *via* UAVs under field conditions against diseases warrants careful consideration, as sub-lethal fungicide exposure may compromise disease control efficacy and increase the risk of fungicide resistance development (Amaradasa and Everhart, 2016). Furthermore, owing to the non-availability of ultra-low-volume (ULV) pesticide formulations in India, the Ministry of Agriculture and Farmers Welfare, Government of India, through the Plant Protection Division has provided interim approval for the application of pesticide formulations already registered for conventional application extended to drones, without changing the active ingredient per acre.

Nonetheless, the successful large-scale adoption of these autonomous systems requires moving beyond the common yet often inadequately validated assumption that a 10 L spray volume per acre and a 25% reduction in fungicide dosage (0.75X) without adjuvants are sufficient for effective control of rice diseases. The application of reduced pesticide dosage and lower spray volume, without understanding the intricacies involved, may contribute to inadequate disease control, non-uniform spray deposition, and the potential development of fungicide-resistant pathogen populations, particularly target-site-based resistance, such as SDH and QoI resistance (Ayer et al., 2020). Further, the challenge of battery endurance compels the operator to adopt lower spray volumes and chemical doses, deviating from established standard operating protocols (SOPs).

The systematic validation of drone-based fungicide application at recommended (1X) and reduced dosages (0.75X) of existing conventional formulations under Indian agro-ecological conditions, particularly for rice blast management, remains limited. Consequently, the credible published literature on fungicide dose optimization for existing conventional formulations and the comparative performance of UAV and conventional spraying methods remains inadequate (Li et al., 2021; Huang et al., 2023; Yallappa et al., 2025). Furthermore, widespread adoption is still constrained by high initial investment costs, a variety of drone design parameters, crop types, lack of technical knowledge, limited battery endurance, shorter flight times (Maddikunta et al., 2021), and large-scale exploitation of non-type certified drones raises serious concerns regarding safety, quality standards, regulatory compliance, and application efficiency.

The equipment-optimized fungicide application is expected to enhance pesticide use efficiency by ensuring effective disease management with reduced inputs and spray volumes (Zhang et al., 2025). In light of the current situation, the present study was conducted through systematic comparative evaluation of fungicide dosage at 1X (100% of the recommended dose) and 0.75X (75% of the recommended dose) of two fungicides *viz.*, Azoxystrobin + Tricyclazole, Azoxystrobin + Difenoconazole, compared to Tricyclazole 75% WP applied using UAVs in comparison with corresponding fungicides applied at the recommended dose (100X) using conventional knapsack power sprayer across two cropping seasons. In addition, the study aimed to evaluate the influence of these treatments on control efficacy (%), phytotoxicity, agronomic yield, and economic viability under field conditions.

## 2 Material and methods

The present study aimed to assess the efficacy of registered conventional fungicides at variable concentrations (1X and 0.75X of the recommended dose) utilizing the UAVs against rice blast under field conditions. The core idea of the present investigation is to evaluate the blast fungicides applied using UAVs, from age-old and cost-effective recommended fungicide *i.e.*, Tricyclazole to new combination fungicide molecules such as Azoxystrobin 18.2% w/w + Difenoconazole 11.4% w/w SC, Azoxystrobin 16.7% + Tricyclazole 33.3% SC, compared with corresponding fungicides applied through Knapsack power sprayer.

### 2.1 Experimental site and design

The field experiment was conducted over two seasons during *kharif*, 2022 and *rabi*, 2022–23 at the Agricultural Research Station (ARS), Kampasagar, Tripuraram mandal, Nalgonda district, Telangana, India. Geographically, the experimental site is located at 15° 32’ N latitude and 76° 34’ E longitude, at an average altitude of 136 m above mean sea level (MSL). According to the modified Troll’s climatic classification, ARS, Kampasagar falls under the semi-arid tropics (SAT), located in the Southern Telangana agro-climatic zone. Agricultural Research Station, Kampasagar, is predominantly cultivated with paddy under assured irrigation. Owing to the requirement for large plot sizes for drone efficacy studies, the extensive paddy ecosystem, favorable weather conditions for paddy cultivation, and its history of consistent natural incidence of blast disease (**Figure 1**), make it ideal for evaluating the effectiveness of drone-based fungicides evaluation against blast.

**Figure 1:**
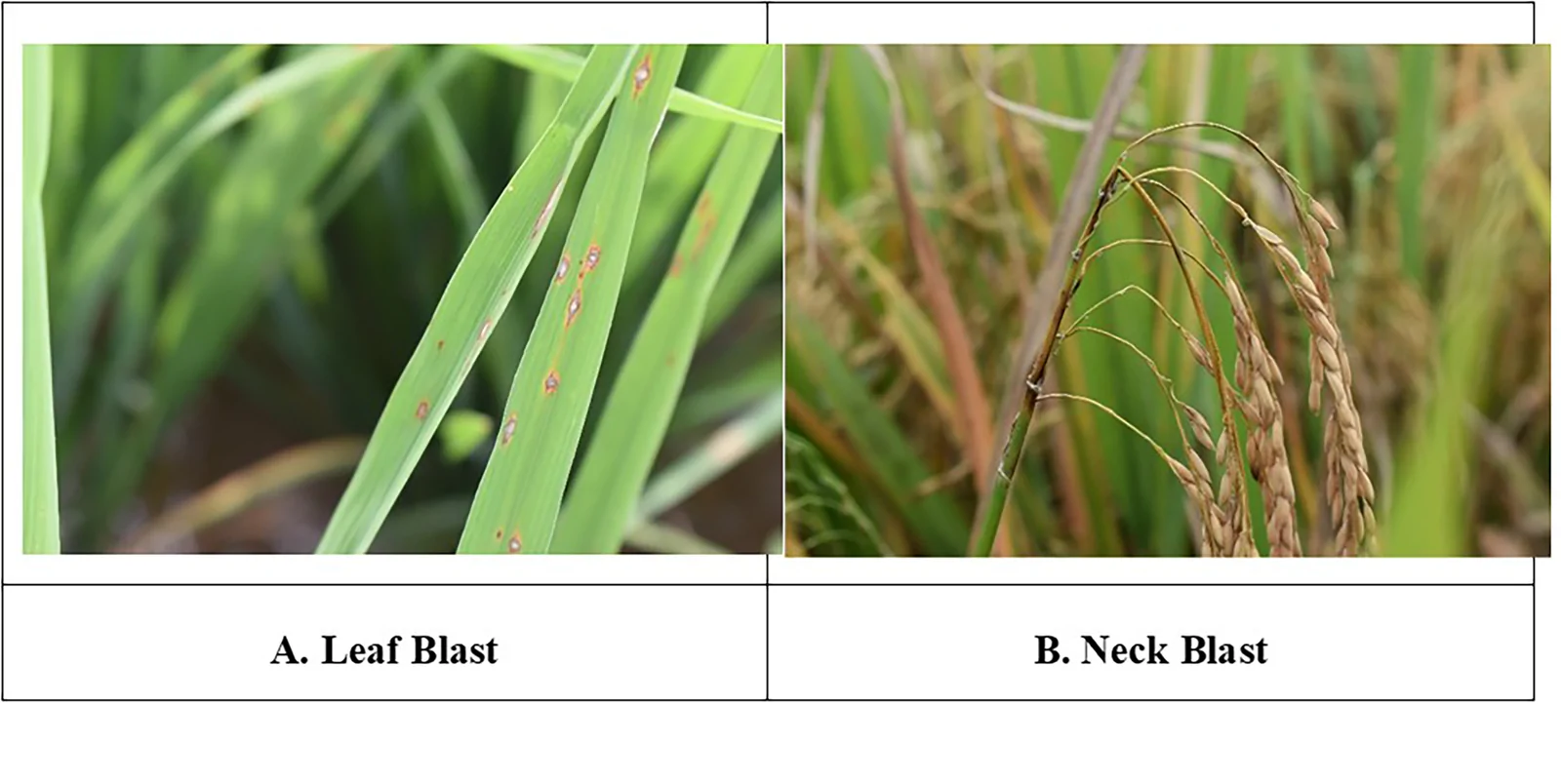
Blast symptoms observed in the experimental field.

The experiment was laid out in a Randomized Complete Block Design (RCBD) with three replications, with each treatment occupying a minimum net plot area of 360 m^2^ per replication to ensure adequate coverage during drone-based pesticide application in rice. While selecting the experimental plots, a minimum size of 26 m length and 14 m width was maintained to ensure sufficient length for continuous and uniform spraying of treatments using drones, and arranged to provide a minimum of 4 drone flying loops (each 3.5 m width), which accounts for approximately 1100 m^2^ for each treatment. This also facilitates achieving the required drone speed in long plots as per SOPs, ensures uniform distribution of spray fluid, and minimizes frequent loops in length and turns during treatment spraying. The selection of larger, elongated plots was intended to mimic actual farmer field conditions and to generate data under realistic large-plot scenarios. A buffer zone of 10 m between plots was maintained to minimize drift and avoid cross-contamination of the spray solution. Further, the data were recorded from the center of each treatment, demarcated as a “valid observation zone” (10 × 8 m) during the first observations, as depicted in **Figure 2**.

**Figure 2:**
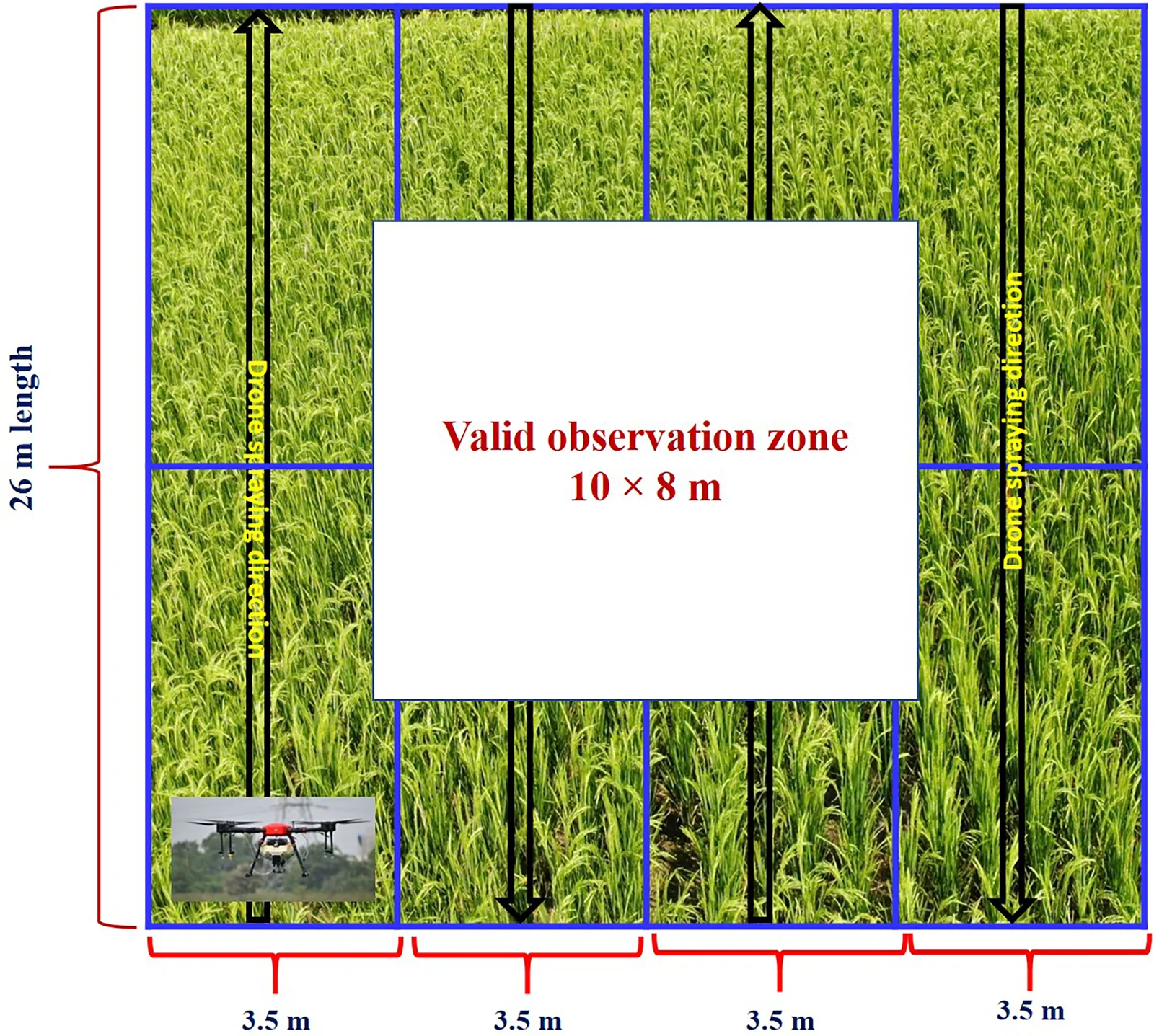
Field layout of each plot depicting drone flying path and observation zone.

The experiment was conducted with Sambha Masuri (BPT 5204), which is highly susceptible to major rice diseases, including blast. The variety was selected for the present investigation because of its continued cultivation over the years, farmer acceptance, market demand, cooking and eating quality, and adaptability across diverse environments. The nursery was sown in raised beds, and 21–25 day-old seedlings were transplanted in the main field. Standard agronomic practices recommended for rice cultivation were uniformly followed across all treatments. The experiment consisted of 10 treatments, including three conventional fungicides applied at two dosage levels designated as “1X” (100%) and “0.75X” (75% of the recommended dose), and their corresponding application using a Knapsack power sprayer at the recommended dose (100%) (**Table 1**). Water spray was used as a control treatment. For instance, the fungicide Tricyclazole 75% WP in a UAV sprayer per hectare for 100% dosage means 300 g of fungicide dissolved in 40 L/ha of water (Varma et al., 2023; Gatkal et al., 2025), whereas 75% dosage means 225 g of Tricyclazole 75% WP dissolved in 40 L of water. For the knapsack power sprayer, 100% dosage means that 300 g of Tricyclazole 75% WP dissolved in 375 L of water (**Table 1**). The leaf blast symptoms were noticed before the first application of fungicides. The fungicides were applied twice per season at PI to the booting stage, with a second spray 20 days after the first spray application.

**Table 1:** Treatment details of the experiment.

Trt. No.	Name of the fungicide	Dosage (%)	Dosage (ha^-1^)	Spraying equipment	Water Volume (L/ha)
T_1_	Azoxystrobin 16.7% + Tricyclazole 33.3% SC	75% (0.75X)	375 mL	Drone	40
T_2_	Azoxystrobin 16.7% + Tricyclazole 33.3% SC	^*^100% (1X)	500 mL	Drone	40
T_3_	Azoxystrobin 16.7% + Tricyclazole 33.3% SC	100% (1X)	500 mL	Knapsack power sprayer	375
T_4_	Azoxystrobin 18.2% w/w + Difenoconazole 11.4 % w/w SC	75% (0.75X)	375 mL	Drone	40
T_5_	Azoxystrobin 18.2% w/w + Difenoconazole 11.4 % w/w SC	100% (1X)	500 mL	Drone	40
T_6_	Azoxystrobin + Difenoconazole 11.4 % w/w SC	100% (1X)	500 mL	Knapsack power sprayer	375
T_7_	Tricyclazole 75% WP	75% (0.75X)	225 g	Drone	40
T_8_	Tricyclazole 75% WP	100% (1X)	300 g	Drone	40
T_9_	Tricyclazole 75% WP	100% (1X)	300 g	Knapsack power sprayer	375
T_10_	Untreated Control	–	–	–	–

* The 100% (1X) dosage is recommended by the Central Insecticide Board & Registration Committee (CIB&RC), Faridabad, Haryana 121 001, India.

### 2.2 Application equipment and operational parameters

The application equipment utilized in the present study comprised a UAV and a Knapsack power sprayer, which was also deployed for comparative analysis (**Figures 3A, B**). The fungicide application was carried out using an agricultural UAV (Marut Drones), which was symmetrically outfitted with flat-fan nozzles (M/s. Spraying Systems Co., Glendale Heights, IL, USA), mounted exactly below the propellers aligned with UAV arms. The detailed technical specifications of the knapsack power sprayer and the UAV used in the present study are mentioned in **Figures 3B** and **4**, respectively. The nozzle selection, flight planning, and operational parameters used for UAV spraying were based on previously developed standard operating procedures (SOPs) for pesticide application using drones (Varma et al., 2023; Ragiman et al., 2024). Earlier, Qin et al. (2016) optimized the flight height to 1.5 m and the flying speed to 5.0 m/sec, achieving maximum lower-layer deposition of pesticides against rice planthoppers. The effectiveness of the TEEJET 11002VS nozzle was also reported in rice (Chen et al., 2020; Guo et al., 2020). Likewise, Liu et al. (2025b), who reported the extended range flat fan nozzle, *i.e.*, XR110015VS (green tip), provided superior droplet density and coverage rates on maize whorls at spray volumes of 37.5 and 45.0 L/ha with a flight height of 2.0 m. Consistency in application treatments and standard parameters was maintained across all the treatments by following the recommended flight parameters and SOPs, outlined in **Figure 5** & **Table 2**. Prior to commencement of the experiment, preliminary calibration tests were performed to optimize UAV sprayer performance and calibrated the discharge rate and nozzle tip pressure using the master application kit and parameters were aligned with the TeeJet product catalog (M/s. Spraying Systems Co., Glendale Heights, IL, USA).

**Figure 3:**
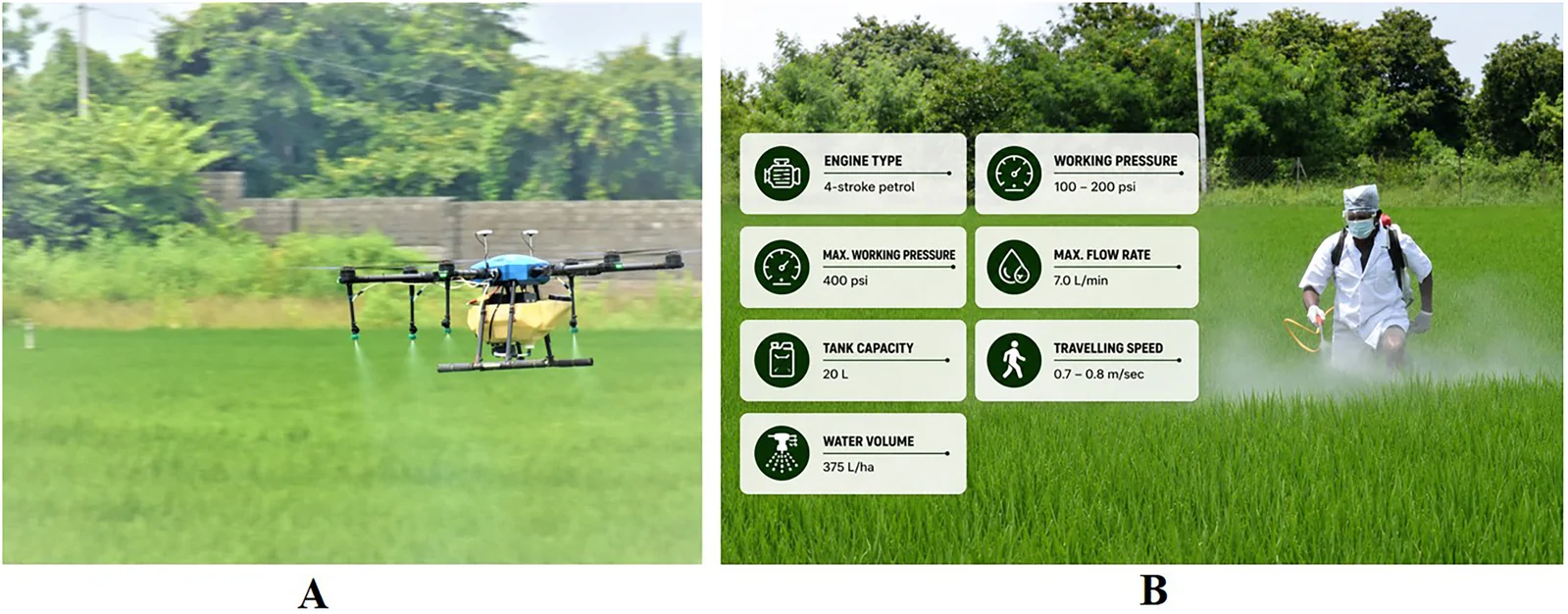
Two test sprayers: **(A)** Six motor uncrewed aerial vehicle; **(B)** Knapsack power sprayer along with specifications.

**Figure 4:**
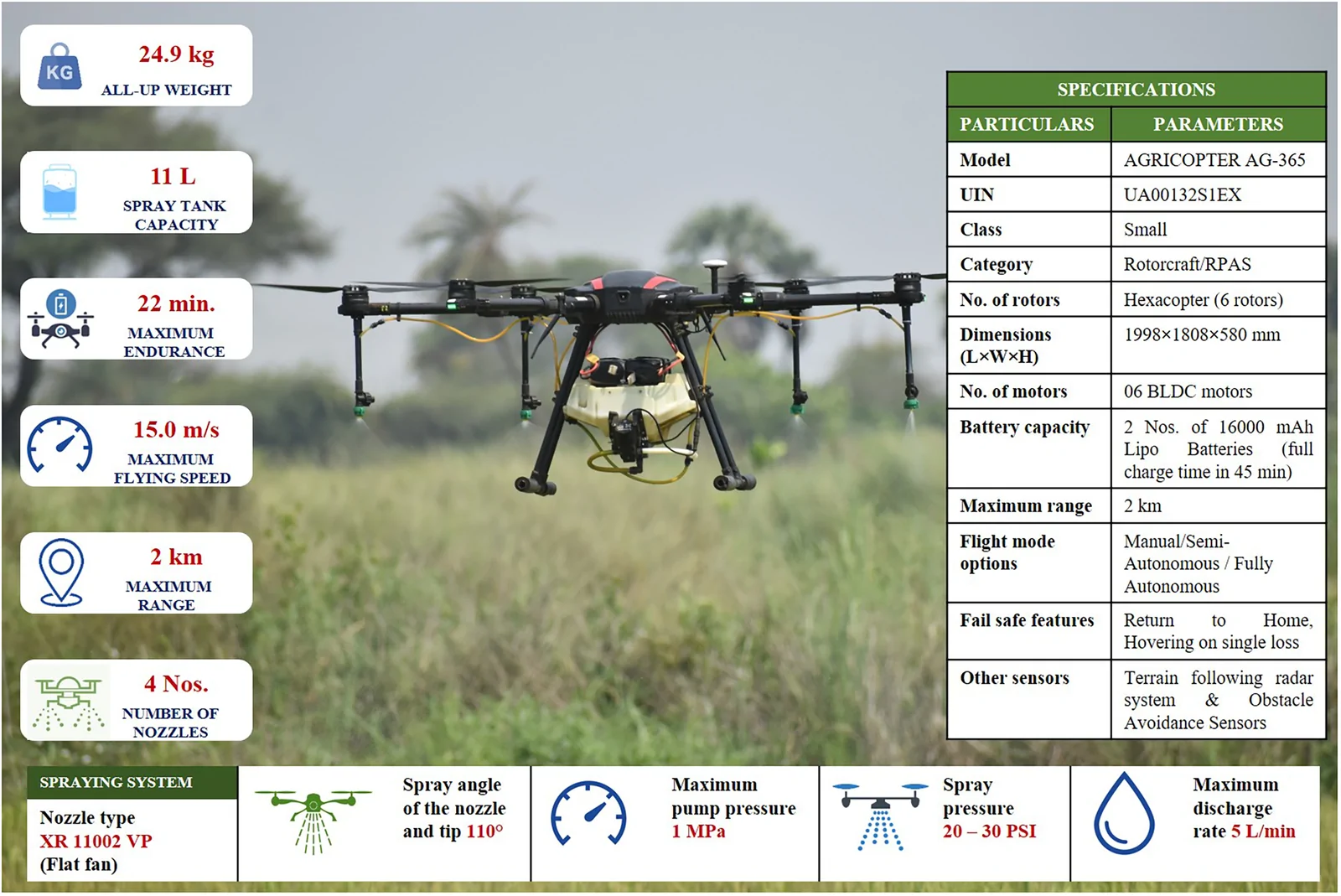
Technical specifications of the hexacopter UAV used for spraying in the experiment.

**Figure 5:**
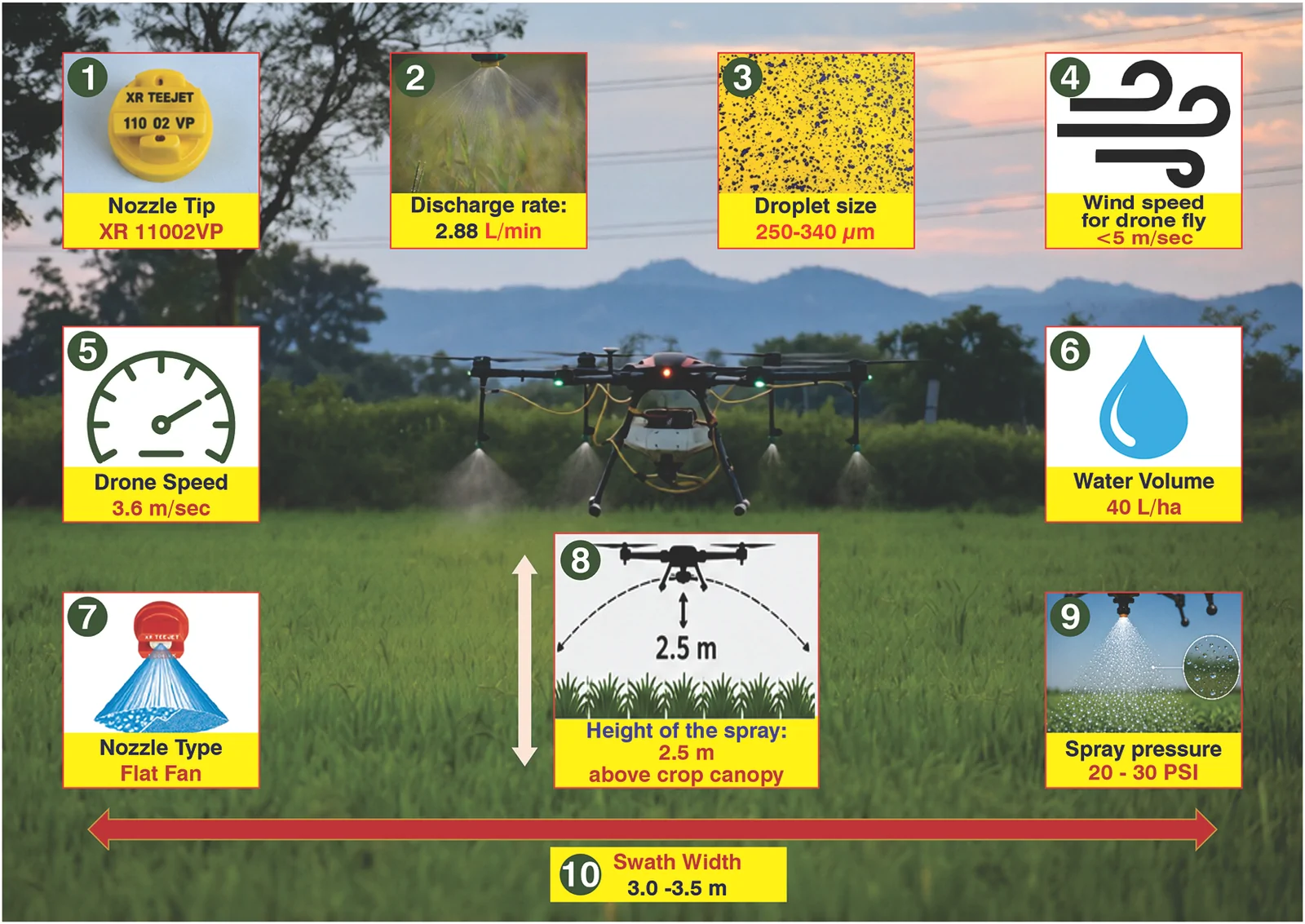
Standard operating parameters followed for UAV spraying during *kharif*, 2022, and *rabi*, 2023–24 at ARS, Kampasagar.

**Table 2:** UAV flight parameters followed for conducting the experiment.

S. no.	Flight parameters	Standard operating procedures
1.	UAV model	AGRICOPTER AG 365
2.	Flight height	2.5 m above the crop canopy
3.	Flight speed	3.6 m/s
4.	Nozzle type	Extended range flat fan
5.	Nozzle tip	XR 11002 VP (Yellow color tip)
6.	Discharge rate	2.88 L/s
7.	Spray width	3 - 3.5 m
8.	Spray pressure	20–30 PSI
9.	Water Volume	40 L/ ha

Uniform application procedures were maintained across all treatments by adhering to the established SOPs and calibrated operating parameters. Prior to application, the experimental treatments were mapped using the Aero GCS Green (https://aerogcs.com) which is a remote controller software. To achieve high spatial accuracy of the field boundaries and precise application of treatments, an orthophotomap for each replication/treatment was created by mapping the boundaries using an agri-drone fitted with an active GPS Neo 3. This mapping enabled uniform treatment spraying in autonomous mode, ensuring repeatability of trajectory, speed, spray height above the crop canopy, and swath width. The spraying activity was accomplished along a preset, mission-planning software-generated flight path rather than by manual piloting. The UAV flight path of one treatment was illustrated in **Figure 6**. The blue line indicates the flying route with starting (S) and final point (F). The drone maintained a pre-defined flight parameters as per the SOPs on rice, while the BLDC pump motor (24 V) was used to pressurize the spray fluid and the intelligent planning operation mode automatically regulated the on-and-off functioning of the spraying system during turns, thereby ensuring stable and uniform distribution of spray droplets across the treatments. This UAV is equipped with a radar-based collision-avoidance and terrain-following sensor positioned directly below the spray tank to maintain altitude in accordance with the crop canopy height. In addition, an obstacle-detection sensor was positioned on the front of the drone to detect obstacles such as transmission lines and electrical poles, thereby preventing collisions and enhancing flight safety. The entire activity is controlled by a well-trained and DGCA-certified remote pilot.

**Figure 6:**
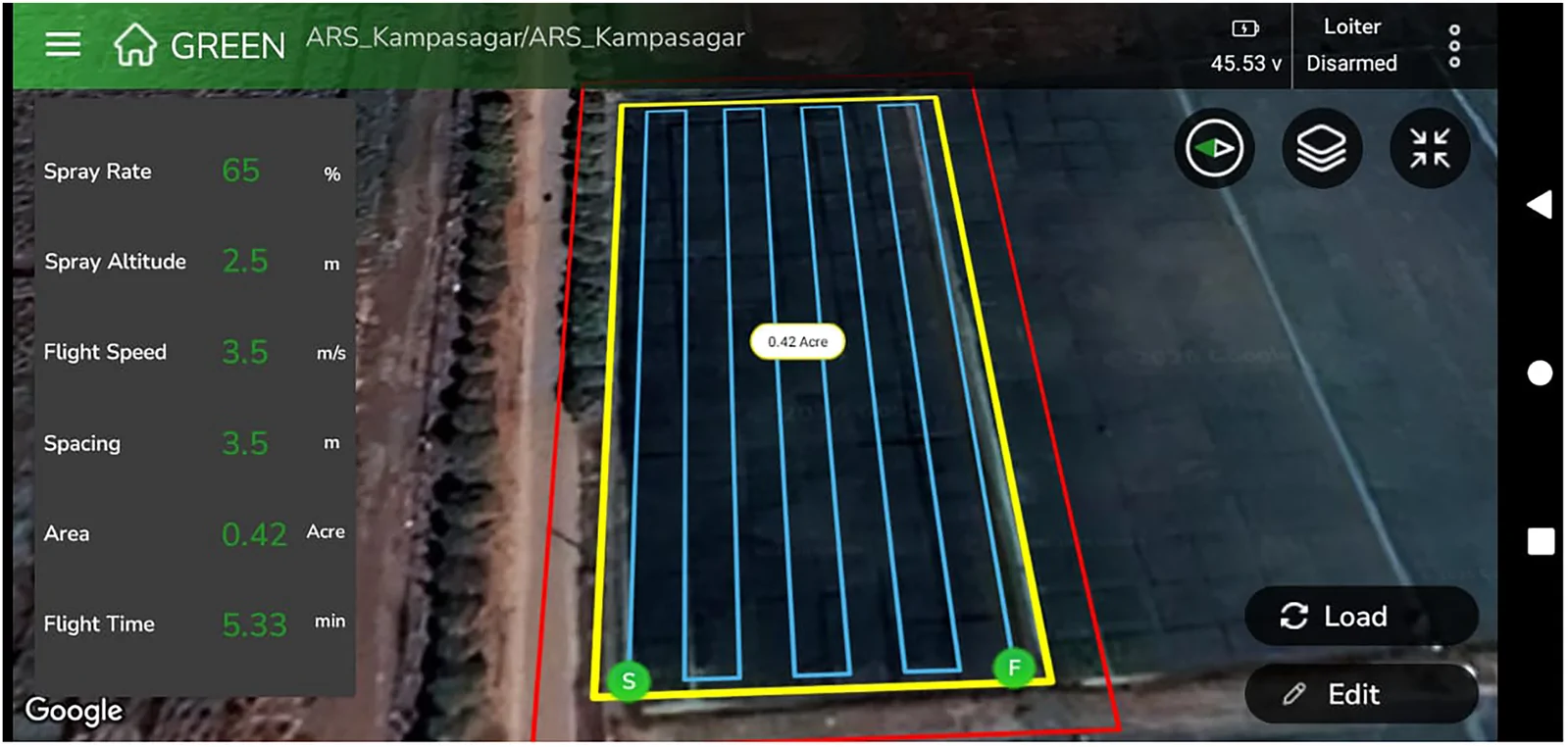
Screen view of the Aero GCS mobile app and UAV flight path of the experiment.

The other conventional sprayer is a knapsack power sprayer (ASPEE EDEN UNIBLAST 4S 31CC with Brass pump, M/s. American Spring & Pressing Works Pvt. Ltd, Mumbai), which was used in the present study, and the detailed specification is outlined in **Figure 3B**. This knapsack power sprayer is equipped with a brass metal pump, 4 stroke petrol engine, tank, spray gun (32”), lightweight and adjustable shoulder straps. The spraying height of the nozzles of the knapsack power sprayer was 0.5 m from the top of the crop canopy.

At the time of treatment application, the climatic conditions, including wind speed, temperature, and relative humidity, were recorded during spraying using a handheld anemometer (Lutron AM-4201) and a hygrometer (HTC 288 CTH) to ensure uniform application conditions. The weather data collected from the Meteorological Observatory, ARS, Kampasagar during the first season of study (*kharif*, 2022) revealed that air temperature ranged from 19.5 to 37.6., while the relative humidity varied between 64.6 to 91.5% and wind speed remained below 1.5 m/sec. In the second season, the air temperature ranged from 18.1 to 37.9 °C, the humidity ranged from 47.1 to 81.8%, and the wind speed ranged from 1.2 to 1.6 m/sec during the experiment. The wind speed and direction were within acceptable ranges during the experiment as per the crop-specific SOPs for the application of pesticides using drones (Ministry of Agriculture & FW, GOI, 2023).

### 2.3 Disease assessment

The leaf blast severity was assessed before fungicide spraying and at 14 days after the first spray using the 0–9 Standard Evaluation System (SES) scale (IRRI, 2013). For recording observations, 20 plants were randomly selected within the valid observation zone for each replication (**Figure 2**), serially numbered, and tagged with red string and plant labels during the first observation stage to ensure consistent monitoring of the same plants throughout the observations.

#### 2.3.1 Leaf blast rating scale

**Table:** Leaf blast rating scale (0 - 9 SES rating scale) followed in the present study.

Scale	Description
0	No lesions
1	Small brown speaks of pinpoint size or larger brown specks without sporulating center.
2	Small roundish to slightly elongated, necrotic gray spots, about 1–2 mm in diameter with a distinct brown margin. Lesions are mostly found on the lower leaves.
3	Lesion type is the same as in scale 2, but a significant number of lesions are on the upper leaves.
4	Typical susceptible blast lesions, 3 mm or longer, infecting less than 4% of the leaf area.
5	Typical blast lesions infecting 4-10% of the leaf area
6	Typical blast lesions infecting 11-25% of the leaf area
7	Typical blast lesions infecting 26-50% of the leaf area
8	Typical blast lesions infecting 51-75% of the leaf area and many leaves dead.
9	More than 75% leaf area affected.

#### 2.3.2 Neck blast rating scale (Incidence of infected panicles)

**Table:** Neck blast rating scale (0 - 9 SES rating scale) followed in the present study.

Scale	Description
0	No incidence
1	Less than 5%
3	5 – 10%
5	11 – 25%
7	26 – 50%
9	More than 50%

The disease ratings were converted into Percent Disease Index (PDI) using the following formula:


$$\text{PDI}(\%)=\frac{\sum \text{Disease ratings}}{\text{Total number of observations}\times \text{Maximum rating}}\times 100$$

Further, the percent reduction over control (PRC) was calculated using the following formula:


$$\text{PRC }(\%)=\frac{\text{C}-\text{T}}{\text{C}}\times 100$$

Where C = PDI in control and T = PDI in treatment.

### 2.4 Phytotoxicity assessment

Phytotoxic effects were assessed at 1, 3, 5, 10, 15, and 20 days after the first spray using a 0–10 rating scale (Rajeswaran et al., 2004). Observations included symptoms such as chlorosis, necrosis, wilting, scorching, epinasty, and hyponasty. Percent phytotoxicity was calculated using standard procedures.

### 2.5 Yield and economic analysis

Grain yield was recorded from each plot and converted to kg ha^-^¹. Economic analysis was performed by calculating the Incremental Cost–Benefit Ratio (ICBR) for each treatment based on grain yield, fungicide cost, spraying equipment, application cost, and the prevailing minimum support price (MSP) for paddy. The cost of operation for UAV spraying (INR 1000 per ha) and the Knapsack power sprayer (INR 800 per ha) was estimated through inquiries with drone operators and farmers from different regions of Telangana to account for variation in local operational rates rather than relying solely on costs at the experimental location.

### 2.6 Statistical analysis

The percentage data is subjected to an angular (arcsine) transformation prior to analysis to stabilize the wide variance and meet the normality assumptions. The experimental and transformed data were subjected to analysis of variance (ANOVA) appropriate for an RCBD, as described by Gomez and Gomez (1984). All treatment means were computed using Analysis of Variance (ANOVA) and compared using Duncan’s Multiple Range Test (DMRT) at a significance level of 95%. The statistical analysis was performed using OPSTAT software (Sheoran et al., 1998) separately for each year and for the pooled data.

## 3 Results

The study evaluated the efficacy of conventional fungicide formulations registered for manual application were tested at the recommended (1X) and 75% of the recommended dosage (0.75X), applied through UAVs, compared with a knapsack power sprayer (1X), against rice blast under field conditions over two crop-growing seasons at ARS, Kampasagar. The control efficacy, yield, phytotoxicity, and economic returns of the knapsack power sprayer were also compared with those of UAV spraying at various fungicide dosages.

The combined analysis of variance revealed highly significant (*P* ≤ 0.01) effects of season, treatment, and season × treatment interaction for leaf blast, neck blast, and grain yield (**Table 3**). The significant seasonal effect indicated variation in disease development and crop productivity between the two growing seasons, while the significant treatment effect confirmed differential responses among fungicide treatments. Furthermore, the significant season × treatment interaction suggested that treatment performance varied across seasons, as confirmed by Bartlett’s test. The results were interpreted and discussed individually for each season.

**Table 3:** Combined analysis of variance for leaf blast, neck blast, and grain yield of rice over two seasons.

Source of variation	df	Leaf blast MS	Neck blast MS	Grain yield MS
Seasons	1	274.7**	1082.9**	21,973,601.7**
Replications within seasons	4	16.047	8.1	473,713.3
Treatments	9	125.6**	188.7**	3,195,803.6**
Seasons × Treatments	9	27.3**	82.4**	824,427.6**
Pooled Error	36	3.6	3.9	363,776.3

Leaf blast and neck blast data were subjected to arcsine square-root transformation prior to analysis. Bartlett's test indicated homogeneity of variances across seasons, permitting pooled analysis. **Significant at *P ≤ 0.01*.

### 3.1 UAV-based fungicide dose optimization against blast during *kharif*, 2022 & *rabi*, 2022-23

As presented in **Table 4**, the efficacy against leaf and neck blast varied significantly across the treatments during *kharif*, 2022 and *rabi*, 2022-23. Leaf blast severity among the treatments ranged from 2.27% to 23.80% during *kharif*, 2022 and from 5.83 to 18.27% during *rabi*, 2022-23. The differences in control efficacy of leaf blast were observed during *kharif*, 2022 following the application of conventional fungicide formulations at the recommended (1X) and reduced dose (0.75X) using UAVs, in comparison with the recommended dosage (1X) applied through a knapsack power sprayer. As illustrated in **Figure 7**, among the UAV treatments, the recommended dose of Azoxystrobin 16.7% + Tricyclazole 33.3% SC and Azoxystrobin 18.2% w/w + Difenoconazole 11.4% w/w SC recorded the highest percent leaf blast reduction over control of 83.7 and 90.5%, respectively, compared with their corresponding reduced dose (0.75X) treatments recorded 76.5 and 71.6% respectively. In contrast, the reduced dose (0.75X) of Tricyclazole 75% WP exhibited comparatively higher numerical control efficacy (83.5%) than its recommended dose applied through UAV (79.8%) and knapsack power sprayer (78.6%).

**Table 4:** Effect of fungicide treatments on leaf blast, neck blast and grain yield of rice during *kharif*, 2022 and *rabi*, 2022–23.

Treatments	Leaf Blast - PDI				Neck Blast - PDI		Yield (Kg/ha)	
	*Kharif*, 2022		*Rabi*, 2022–23		*Kharif*, 2022	*Rabi*, 2022–23	*Kharif*, 2022	*Rabi*, 2022–23
	PRC	14 DAS	PRC	14 DAS				
T_1_: Azoxystrobin 16.7% + Tricyclazole 33.3% SC @ 0.75X with UAV	15.62 (23.15) ^ab^	5.60 (13.67) ^bc^	5.50 (13.54) ^c^	6.93 (15.23) ^d^	27.17 (31.43) ^ef^	26.10 (30.73) ^b^	5483 ^ab^	4577 ^a^
T_2_: Azoxystrobin 16.7% + Tricyclazole 33.3% SC @ 1X with UAV	17.94 (24.80) ^abc^	3.87 (10.90) ^bc^	3.11 (10.15) ^b^	5.83 (13.93) ^d^	7.87 (16.00) ^g^	8.09 (16.21) ^e^	6150 ^a^	4550 ^a^
T_3_: Azoxystrobin 16.7% + Tricyclazole 33.3% SC @ 1X with Knapsack power sprayer	17.71 (24.81) ^abc^	4.03 (11.53) ^bc^	1.19 (6.24) ^a^	7.37 (15.77) ^d^	30.30 (33.37) ^de^	24.37 (29.53) ^b^	5823 ^ab^	3350 ^b^
T_4_: Azoxystrobin 18.2% w/w + Difenoconazole 11.4 % w/w SC @ 0.75X with UAV	13.22 (21.26) ^a^	6.77 (15.00) ^b^	3.49 (10.76) ^b^	9.63 (18.07) ^bc^	42.40 (40.63) ^b^	12.37 (20.60) ^de^	5017 ^ab^	3633 ^b^
T_5_: Azoxystrobin 18.2% w/w + Difenoconazole 11.4 % w/w SC @ 1X with UAV	17.17 (24.28) ^ab^	2.27 (7.63) ^c^	1.32 (6.60) ^a^	6.20 (14.43) ^d^	26.10 (30.73) ^ef^	11.90 (20.20) ^de^	5117 ^ab^	4650 ^a^
T_6_: Azoxystrobin 18.2% w/w + Difenoconazole 11.4 % w/w SC @ 1X with knapsack power sprayer	19.33 (26.03) ^bcd^	2.37 (8.67) ^c^	3.12 (10.17) ^b^	7.40 (15.77) ^d^	39.47 (38.90) ^bc^	19.23 (26.00) ^c^	5423 ^ab^	3183 ^b^
T_7_: Tricyclazole 75% WP @ 0.75X with UAV	23.40 (28.91) ^cd^	3.93 (10.90) ^bc^	0.87 (5.19) ^a^	7.63 (16.03) ^cd^	34.07 (35.67) ^cd^	16.20 (23.73) ^cd^	4300 ^bc^	3533 ^b^
T_8_: Tricyclazole 75% WP @ 1X with UAV	16.19 (23.71) ^ab^	4.80 (12.47) ^bc^	1.62 (7.31) ^a^	7.33 (15.73) ^d^	22.57 (28.30) ^f^	13.67 (21.70) ^d^	4950 ^ab^	4733^a^
T_9_: Tricyclazole 75% WP @ 1X with Knapsack power sprayer	15.52 (23.20) ^ab^	5.10 (12.46) ^bc^	1.09 (5.27) ^a^	11.30 (19.60) ^b^	36.17 (36.93) ^bcd^	15.20 (22.93) ^cd^	6033 ^a^	4683 ^a^
T_10_: Untreated Control	25.54 (30.33) ^d^	23.80 (29.17) ^a^	7.18 (15.53) ^d^	18.27 (25.17) ^a^	50.87 (45.53) ^a^	33.33 (35.23) ^a^	3300 ^c^	2600 ^c^
SEm (±)	1.79	1.14	0.27	0.73	1.34	1.5	463.47	166.48
CD (*P* = 0.05)	5.31	3.38	0.81	2.17	3.98	4.5	1387.71	498.46
CV (%)	17.0	17.7	9.8	7.43	6.87	14.5	15.56	7.30

Values outside parentheses represent the original PDI values, whereas values in parentheses represent the arcsine square-root transformed values used for statistical analysis and DMRT. Means followed by the same letter(s) within a column are not significantly different according to Duncan’s Multiple Range Test (DMRT) at *P* ≤ 0.05; PRC: Pre-count of disease (baseline disease severity).

**Figure 7:**
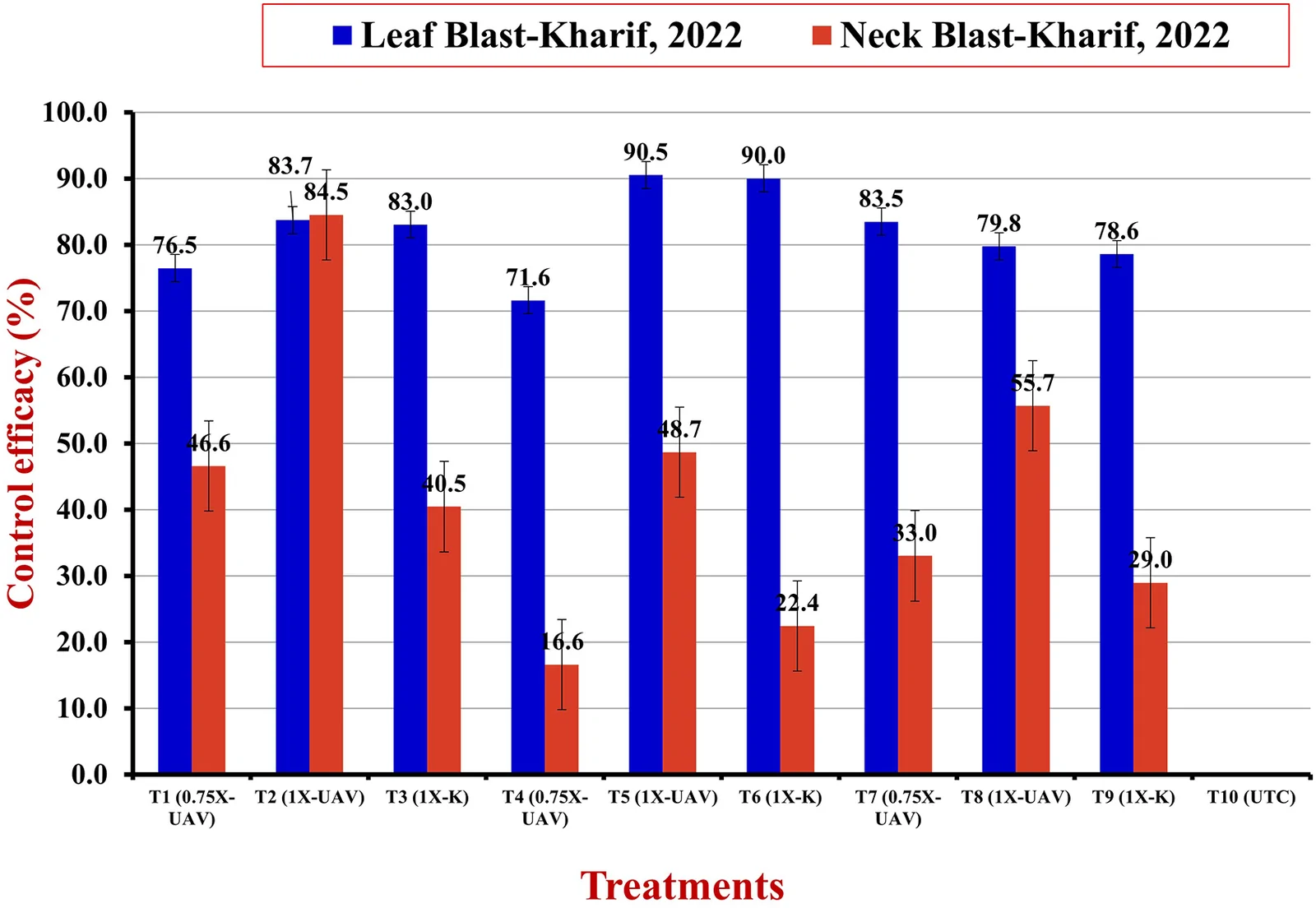
Control efficacy of nine experimental fungicide dosages applied using UAV and Knapsack Power Sprayer (K) against rice blast during *kharif*, 2022.

Among the treatments, the variations in control efficacy of leaf blast during *rabi*, 2022-23, ranged from 38.1 to 68.1% (**Figure 8**). The highest leaf blast control efficacy of 68.1% was achieved with UAV application of Azoxystrobin 16.7% + Tricyclazole 33.3% SC @ 1X, followed by Azoxystrobin 18.2% w/w + Difenoconazole 11.4% w/w SC (66.0%) at 1X-UAV, whereas the lowest control efficacy was exhibited by manual spraying of Tricyclazole 75% WP at 1X dosage. It is pertinent to mention that leaf blast severity during *rabi*, 2022-23, was relatively lower than that observed during *kharif*, 2022. Although leaf blast is typically more severe during the *rabi* season in Telangana state, comparatively lower disease incidence was witnessed due to variations in temperature, relative humidity, leaf wetness duration, and rainfall distribution. Further, the leaf blast severity was assessed at 14 days after the first spray of fungicides, while a second spray was applied 20 days later. However, no separate quantitative assessment of leaf blast was conducted after the second spray, because leaf blast progression from flowering to the milky stage was very low, and the prevailing severity was insufficient to reliably discriminate treatment effects. In the present study, the leaf blast efficacy data primarily represent the response to the first fungicidal spray rather than the cumulative efficacy of two sprays warranting further validation with post-second-spray assessments for a complete line of evidence.

**Figure 8:**
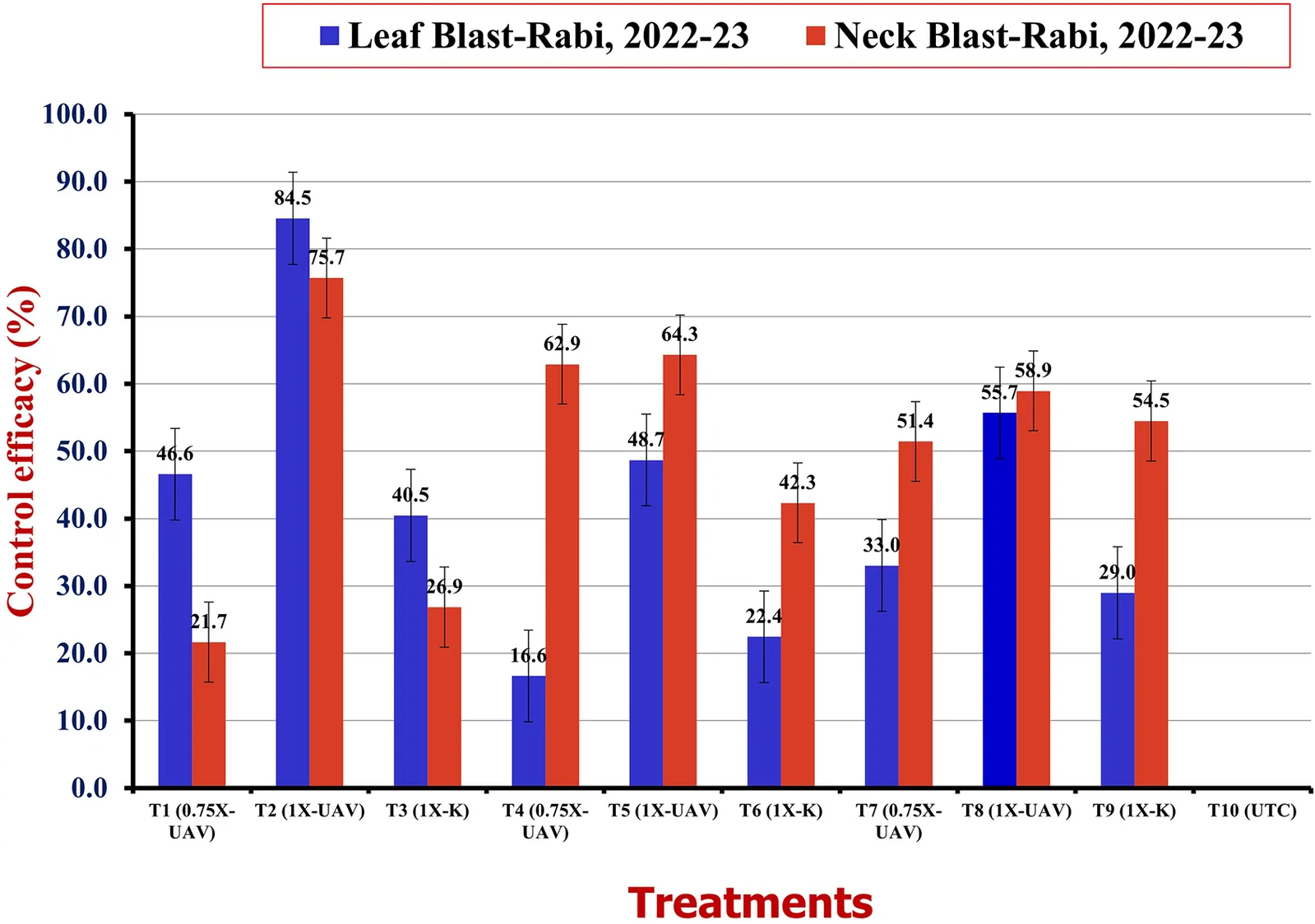
Control efficacy of fungicide dosages applied using UAV and Knapsack power sprayer against rice blast during *Rabi*, 2022-23.

The neck blast PDI across treatments ranged from 7.87% to 50.87% during *kharif*, 2022. Among the UAV treatments, spraying at the recommended dosage (1X) consistently recorded the lowest neck blast severity, ranging from 7.87% to 26.10%, and differed significantly from the corresponding reduced-dose (0.75X) and conventional knapsack power spraying treatments. In contrast, the reduced-dose UAV applications (0.75X) recorded neck blast severity ranging from 27.17 to 42.40%, which, although significantly lower than the untreated control (50.87%). The application of the same fungicides using a knapsack power sprayer resulted in neck blast severity ranging from 30.30 to 39.47%. The percent reduction of neck blast over the control during *kharif*, 2022 ranged from 16.6 to 84.5. Moreover, the control efficiency of UAV-based fungicide spraying treatments at the 1X dose ranged between 48.7 to 84.5%, whereas at 0.75X dose varied between 16.6 to 46.6%. For instance, the UAV spraying of Azoxystrobin 16.7% + Tricyclazole 33.3% SC @ 1X dose achieved a control efficiency of 84.5%, compared with 46.6% at reduced dosage (0.75X) and 40.5% when applied through manual knapsack power sprayer.

During *rabi*, 2022-23, the neck blast PDI among fungicide treatments, irrespective of application method, ranged from 8.09 to 26.10% compared with the untreated control (33.33%). This corresponds to a per cent reduction over the control, ranging from 75.7 to 21.7%. The lowest neck blast severity (8.09%) was achieved with Azoxystrobin 16.7% + Tricyclazole 33.3% SC at 1X dose, which is statistically equivalent to Azoxystrobin 18.2% w/w + Difenoconazole 11.4% w/w SC applied through UAVs, at both 1X and 0.75X dosages. However, a significant difference was observed between the 1X dose of Azoxystrobin 16.7% + Tricyclazole 33.3% SC, *vis-à-vis* the 0.75X and 1X doses of conventional manual spraying. Notably, no significant difference was observed between this fungicide at 0.75X-UAV and the 1X dose applied with a knapsack sprayer, despite changes in the quantity of active ingredient applied. Similar treatment responses were observed for per cent reduction over control during *rabi*, 2022-23, except Azoxystrobin 16.7% + Tricyclazole 33.3% SC, which showed a profound difference in neck blast control efficacy at 1X-UAV (75.7%) outperforming both 0.75X-UAV (21.7%) and 1X-manual (26.9%) application of the same fungicide. Overall, a consistent trend was observed in neck blast control efficacy across fungicides, indicating that UAV spraying at the recommended dosage (1X) achieved higher control efficacy than the corresponding reduced-dose application (0.75X) and conventional knapsack spraying at the recommended dosage (1X) of conventional fungicide formulations.

### 3.2 Assessment of phytotoxicity, grain yield, and incremental cost-benefit ratio during *kharif*, 2022 and *rabi*, 2022-23

Phytotoxicity studies indicated that none of the fungicide treatments induced any phytotoxicity symptoms such as yellowing, leaf tip drying, or vein clearing at any of the observed time points (1, 3, 5, 10, 15, and 20 days after the first spray). These findings confirm that the fungicide treatments were safe for rice plants and did not cause adverse effects when applied through UAVs at either the recommended (1.0X) or reduced (0.75X) dosage.

During *kharif*, 2022, the treatments T2 (Azoxystrobin + Tricyclazole) at 1X dosage applied with a UAV, and T9 (Tricyclazole) at 1X applied with Knapsack power sprayer exhibited the highest grain yield of 6150 and 6033 kg/ha, respectively. These treatments were statistically comparable to the other fungicide treatments but significantly superior to the untreated control. The lowest grain yield (3300 kg/ha) in the untreated control signifies the role of effective blast management in rice in enhancing productivity. Furthermore, economic analysis based on the incremental cost-benefit ratio (ICBR) revealed that, owing to superior yield performance and relatively low input costs, manual application of Tricyclazole (T9) has recorded the highest ICBR of 1:13.5, which was followed by T2 (Azoxystrobin + Tricyclazole) exhibited 1:8.6 ICBR and the remaining treatments registered ICBR values ranging from 1:3.2 to 1:7.9.

In *rabi*, 2022-23, the best fungicide treatment, such as UAV spraying of Azoxystrobin + Tricyclazole (T2) at the recommended dosage, recorded a grain yield of 4550 kg/ha, which is statistically equivalent to other UAV-applied fungicide treatments (T1, T5, T7, and T8). The ICBR varied from 0.4 to 10.3 across the treatments. Like *kharif* season, Tricyclazole applied through UAV (T8) and knapsack sprayer (T9) at recommended dose has generated comparatively higher economic returns, exhibiting ICBR values of 1:10.3 and 1:10.0, respectively (**Figure 9**). The cost-effectiveness and operational costs may vary among regions and scale of operation with field size, service availability, operational efficiency, availability of trained operators, labor rates, and prevailing input prices. Therefore, the reported ICBR values may be interpreted as period- and region-specific estimates and extrapolated to other regions or operational scales with due consideration of prevailing costs and market conditions.

**Figure 9:**
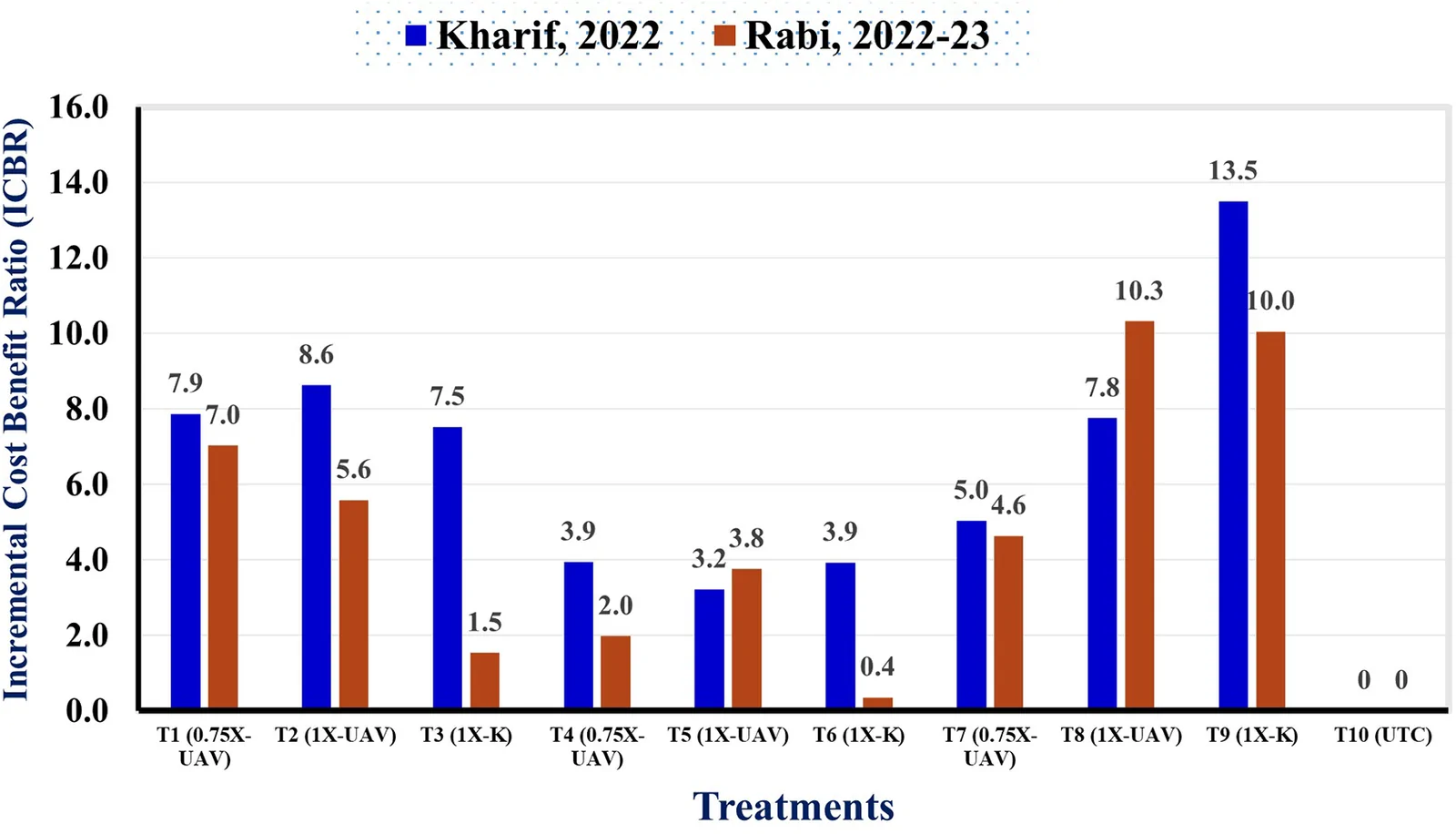
Incremental cost-benefit ratio of various fungicide dosages applied using UAVs against blast of rice.

## 4 Discussion

The superior disease suppression observed with UAV application should be interpreted cautiously, as UAV (40 L ha^-^¹) and knapsack spraying (375 L ha^-^¹) differed substantially in spray volume and spray-solution concentration in addition to application equipment. These differences may influence droplet behavior, deposition, retention, and canopy coverage; therefore, the observed equipment effect cannot be attributed solely to the UAV platform. Likewise, the reduced-dose UAV treatment (0.75X) combined with lower water volume (40 L/ha) may have resulted in decrease in the absolute amount of active-ingredient deposition and lower coverage on the plant surface, particularly under higher disease pressure (Wang et al., 2023; Weicai and Panyang, 2023; Bonds et al., 2024). The use of reduced fungicide doses has been discussed in the literature as a potential factor influencing resistance-selection pressure in pathogen populations, particularly target-site resistance (Ayer et al., 2020). Nonetheless, Liu et al. (2025a) found that orysastrobin and flutolanil showed greater sheath blight control efficacy at 1/2X and 1/4X concentrations than at 1X when applied using mobile spraying equipment. The authors attributed this response to several complex factors, including the crop growth stage and the potential negative effects that a recommended dosage might have on the plants. However, these findings were generated under controlled indoor simulation conditions designed to optimize application parameters and may not be directly extrapolated to field conditions, where environmental and operational factors substantially influence UAV spray performance and fungicide efficacy.

In contrast, manual spraying in waterlogged rice fields is often hampered by operator mobility constraints, physical drudgery, operator-related application variability, and the requirement of large spray volumes and dilution, all of which may reduce fungicide efficacy (Punyawattoe et al., 2021; Jeevan et al., 2023). Furthermore, conventional pesticide application methods may not always enable timely disease management during rice blast outbreaks (Cao et al., 2021).

The effect of dose reduction studies demonstrated that, UAV-based pesticide spraying can reduce chemical pesticide usage by 20-30% while maintaining effective pest and disease control against wheat aphids (Meng et al., 2018), wheat aphids (Wang et al., 2019), in sugarcane against stem borer (Zhang et al., 2019); aphids and Phytophthora blight in pepper (Xiao et al., 2020), rice leaf roller (Wang et al., 2023), garlic leaf blight (Park et al., 2024) and in tea against leafhoppers (Wu et al., 2025) and concluded that, the feasibility of dose reduction is highly dependent on weather conditions, pesticide formulations, operational parameters, crop architecture, target organism, and application technology. Consequently, the suitability of dose-reduction strategies for conventional fungicide formulations under Indian rice production systems remains uncertain and warrants further investigation. There are contrasting views on the existing conventional pesticide concentrations when using UAVs across various crops. One perspective suggests that repeated exposure to sub-lethal doses may accelerate the development of resistant populations, particularly target-site resistance, while the other is that a 20-30% reduction in pesticide concentration can be achieved without compromising biological efficacy under optimal spray parameters, when conventional fungicide formulations are supplemented with suitable adjuvants.

A review of available literature on the comparison between the low-volume UAV application of fungicides at recommended and reduced dosages to the high-volume knapsack sprayer at the recommended dose reveals contrasting findings regarding disease control efficacy across various crops. For instance, a study on evaluating reduced dose of triadimefon 44%SC without adjuvants at 0.6X (270 g/ha) and 0.8X (360 g/ha) compared with 1X-UAV (450 g/ha) and 1X-Knapsack sprayer (450 g/ha) against powdery mildew of wheat revealed that, the control efficacy of 1X-UAV (68%) significantly lower than the knapsack sprayer (73%) at ten days after application (Qin et al., 2018). In contrast, other studies have demonstrated that low-volume UAV-based pesticide application, when combined with suitable adjuvants, reduces the required pesticide dosage without sacrificing efficacy against leaf blast (Wang et al., 2024). These contrasting findings indicate that the pesticide dose reduction under UAV-based application is influenced by multiple factors, including pesticide formulation, active ingredient, spray volume, crop architecture, canopy density, target organism, tank mixing of adjuvants and spray operational parameters. In general, effective management of fungal diseases is more challenging, as UAV application of conventional fungicide formulations may be influenced by canopy coverage and penetration into infection sites, which are essential compared to insect-pests, where pest mobility often reduces dependence on good coverage (Bonds et al., 2024).

The present study demonstrated that the response of rice blast to fungicide application varied with fungicide dosage and application equipment. In UAV applications, the recommended dosage (1X) of conventional fungicide formulations provided more consistent suppression of both leaf and neck blast than the corresponding reduced dosage (0.75X), with the difference more pronounced for neck blast. This indicates that direct reduction of the dosage of conventional fungicide formulations may not consistently maintain adequate disease suppression under the tested field conditions. The comparatively greater sensitivity of neck blast to dose reduction is particularly important because infection at the panicle and neck region can directly affect grain formation and yield.

## 5 Conclusion

The present study represents one of the first systematic field-based evaluation in India to evaluate direct fungicide dose reduction under UAV-assisted application for rice blast management, using currently registered conventional formulations without adjuvant supplementation. Across two cropping seasons, UAV application at the recommended dosage (1X) provided more consistent suppression of leaf and neck blast than the corresponding reduced dosage (0.75X) and conventional knapsack spraying (1X), with the effect being more pronounced for neck blast under field conditions. The findings indicate that an arbitrary reduction of currently registered fungicide dosages under UAV application may compromise blast control in rice under field conditions. The study has provided a preliminary field evaluation under specific agro-ecological, varietal, and operational conditions and basis of potential scalability for similar research in other rice-growing regions or varieties.

Furthermore, the results highlight the need for crop, disease, formulation-specific field validation before recommending fungicide dose reduction, as prolonged exposure to sub-lethal fungicide doses may increase the selection pressure and potentially accelerate fungicide resistance development. Future investigations involving evaluation at multiple locations, seasons, varieties with varying levels of blast resistance, spray-deposition measurements and drone-ready formulations along with suitable adjuvants are warranted to determine the broader feasibility of fungicide dose optimization under UAV-based applications for management of rice blast.

## Data Availability

The raw data supporting the conclusions of this article will be made available by the authors, without undue reservation.
