# Supplementary material for: UAV-based application of fungicides for management of blast disease in rice: comparative evaluation with manual spraying at recommended and reduced doses
Source: Front Plant Sci. 2026 Oct 30;17:1936875. doi: 10.3389/fpls.2026.1936875 (PMC13759828; doi:10.3389/fpls.2026.1936875)
Supplement: Supplementary file 1 [file Table1.docx]

**SUPPLEMENTARY TABLE 1. Incremental Cost Benefit Ratio (ICBR) of various treatments during *kharif*, 2022**

| **Trt. No.** | **Grain yield**  **(q/ha)** | **Incremental yield over control (q/ha)** | **Incremental Cost (INR/ha)** | **Incremental returns over control (INR/ha)** | **ICBR** |
| --- | --- | --- | --- | --- | --- |
| T1 | 54.83 | 21.83 | 5075 | 44977 | 7.9 |
| T2 | 61.50 | 28.50 | 6100 | 58710 | 8.6 |
| T3 | 50.17 | 17.17 | 7159 | 35363 | 3.9 |
| T4 | 51.17 | 18.17 | 8878 | 37423 | 3.2 |
| T5 | 43.00 | 10.00 | 3413 | 20600 | 5.0 |
| T6 | 49.50 | 16.50 | 3884 | 33990 | 7.8 |
| T7 | 58.23 | 25.23 | 6100 | 51981 | 7.5 |
| T8 | 54.23 | 21.23 | 8878 | 43741 | 3.9 |
| T9 | 60.33 | 27.33 | 3884 | 56307 | 13.5 |
| T10 | 33.00 | 0.0 | 0.0 | 0.0 | 0.0 |

Note: Minimum Support Price of paddy = 2060/INR per quintal,

Cost of Azoxystrobin 16.7% + Tricyclazole 33.3% SC (500 ml/ha): INR 2050/-, Azoxystrobin 18.2% w/w + Difenoconazole 11.4 % w/w SC (500 ml/ha): INR 3439/-; Tricyclazole 75% WP (300 g/ha): INR 942/-; Spraying cost (ha): Taiwan sprayer spraying charges = INR.800/- & Drone spraying charges = INR.1000/-.

**SUPPLEMENTARY TABLE 2. Incremental Cost Benefit Ratio (ICBR) of various treatments during *rabi*, 2022-23**

| **Trt. No.** | **Grain yield**  **(q/ha)** | **Incremental yield over control (q/ha)** | **Incremental Cost (INR/ha)** | **Incremental returns over control (INR/ha)** | **ICBR** |
| --- | --- | --- | --- | --- | --- |
| T1 | 45.77 | 19.77 | 5075 | 40719 | 7.0 |
| T2 | 45.50 | 19.50 | 6100 | 40170 | 5.6 |
| T3 | 36.33 | 10.33 | 7159 | 21287 | 2.0 |
| T4 | 46.50 | 20.50 | 8878 | 42230 | 3.8 |
| T5 | 35.33 | 9.33 | 3413 | 19227 | 4.6 |
| T6 | 47.33 | 21.33 | 3884 | 43947 | 10.3 |
| T7 | 33.50 | 7.50 | 6100 | 15450 | 1.5 |
| T8 | 31.83 | 5.83 | 8878 | 12017 | 0.4 |
| T9 | 46.83 | 20.83 | 3884 | 42917 | 10.0 |
| T10 | 26.00 | 0.00 | 0.0 | 0 | 0.0 |

Note: Minimum Support Price of paddy = 2060/INR per quintal,

Cost of Azoxystrobin 16.7% + Tricyclazole 33.3% SC (500 ml/ha): INR 2050/-, Azoxystrobin 18.2% w/w + Difenoconazole 11.4 % w/w SC (500 ml/ha): INR 3439/-; Tricyclazole 75% WP (300 g/ha): INR 942/-; Spraying cost (ha): Taiwan sprayer spraying charges = INR.800/- & Drone spraying charges = INR.1000/-.
